# Analysis of Novel Mycobacteriophages Indicates the Existence of Different Strategies for Phage Inheritance in Mycobacteria

**DOI:** 10.1371/journal.pone.0056384

**Published:** 2013-02-28

**Authors:** Emma J. Stella, Jorgelina J. Franceschelli, Sabrina E. Tasselli, Héctor R. Morbidoni

**Affiliations:** Cátedra de Microbiología, Facultad de Ciencias Médicas, Universidad Nacional de Rosario, Rosario, Argentina; Rockefeller University, United States of America

## Abstract

Mycobacteriophages have been essential in the development of mycobacterial genetics through their use in the construction of tools for genetic manipulation. Due to the simplicity of their isolation and variety of exploitable molecular features, we searched for and isolated 18 novel mycobacteriophages from environmental samples collected from several geographic locations. Characterization of these phages did not differ from most of the previously described ones in the predominant physical features (virion size in the 100–400 nm, genome size in the 50–70 kbp, morphological features compatible with those corresponding to the Siphoviridae family), however novel characteristics for propagation were noticed. Although all the mycobacteriophages propagated at 30°C, eight of them failed to propagate at 37°C. Since some of our phages yielded pinpoint plaques, we improved plaque detection by including sub-inhibitory concentrations of isoniazid or ampicillin-sulbactam in the culture medium. Thus, searches for novel mycobacteriophages at low temperature and in the presence of these drugs would allow for the isolation of novel members that would otherwise not be detected. Importantly, while eight phages lysogenized *Mycobacterium smegmatis*, four of them were also capable of lysogenizing *Mycobacterium tuberculosis*. Analysis of the complete genome sequence obtained for twelve mycobacteriophages (the remaining six rendered partial genomic sequences) allowed for the identification of a new singleton. Surprisingly, sequence analysis revealed the presence of *par*A or *par*A/*par*B genes in 7/18 phages including four that behaved as temperate in *M. tuberculosis*. In summary, we report here the isolation and preliminary characterization of mycobacteriophages that bring new information to the field.

## Introduction

With over two million deaths per year worldwide, *Mycobacterium tuberculosis,* the pathogen responsible for human tuberculosis, is one of the deadliest human pathogens especially when associated to AIDS or other health situations that reduce the capability of the immune system [1]. To this death toll the burden of economic losses (job losses, social segregation, and supervision of special programs for treatment) add up to make tuberculosis a still undefeated global public health enemy. There are few therapeutic options for tuberculosis treatment and clinical strains resistant to one or several of the anti-tubercular drugs have been described [2]–[4]. In order to improve existing drugs or to develop new ones, an understanding of mycobacterial physiology and pathogenesis mechanisms is urgently needed. Both fields have been lagging behind more developed ones, such as immunology of tuberculosis, mainly due to the lack of appropriate genetic tools to manipulate the tubercle bacilli. Mycobacteriophages were already described and used for species typing [5]–[7] but their use bloomed with the pioneering work carried on by the groups of Hatfull and Jacobs in USA, which led to the construction of transposon delivery vectors, cloning vectors and later to the development of an specialized transduction system for generation of gene knock-outs, all of them phage-based tools [8], [9]. Therefore, the last fifteen years witnessed an exponential increase in the research on major topics such as the identification of the molecular factors involved in virulence, targets for anti-tubercular and anti-mycobacterial drugs as well as identification of the components of mycobacterial pathways [10]–[15]. However, although the utilization of mycobacteriophages as the scaffold of novel tools for genetic manipulation of mycobacteria allowed major breakthroughs on the physiology and pathogenesis of those microorganisms, it is on the field of phage genetics and evolution where the paramount work of Hatfull’s group becomes the foundation of our knowledge of mycobacteriophages. Over the last two decades, his group isolated and characterized over 3,300 mycobacteriophages, with more than 10% of them sequenced and over 200 fully annotated as mentioned in his Phage Database webpage (www.phagesdb.org.) While doing that, this group developed bioinformatics tools such as Phamerator, a program used for comparative genomic analysis by assembling protein coding genes into families (dubbed as *Pha*milies) and thus making simple and fast the comparison of mycobacteriophage proteins [16]. The analysis of such a large number mycobacteriophage genomes rendered several features of interest from the point of view of evolution, such as the mosaicism of their genes [17], [18], and the presence of ultra small insertion sequences detected in mycobacteriophages Halo, Hope and BPs [19], [20]. As an example of the tremendous potential of mycobacteriophage genomics we may mention the studies focusing on the integration-excision systems that have been so important at the time of constructing vectors for the integration of extra copies of genes of interest in the mycobacterial chromosome [21], [22]. On the other hand, controlled expression of excisionases led to loss of those integrated copies going back to the previous phenotype [23]. More recently, research on the mechanisms leading to phage release identified two endolysins, designated as LysA and LysB with hydrolytic specificity for peptidoglycan and mycoloylarabinose respectively [24], [25]. Regulated expression of those enzymes –along with the cognate holin needed to allow LysA and LysB translocation outside the mycobacterial cell- could be of potential interest to efficiently achieve cell lysis of *M. tuberculosis* without requiring costly equipment. Last but not least, the finding of novel recombinogenic enzymes in mycobacteriophage Che9 led to the development of a novel system for the precise deletion of chromosomal genes [26], [27]; even more the introduction of point mutations in genes of interest would allow for constructing a library of alleles to test for defined phenotypes [28], [29]. Thus, this technique could be applied to the identification of aminoacid residues of importance for protein stability or domain structural definition in novel gene products for which such information is not readily available. The outstanding body of information produced by Hatfull’s group by the analysis of a large number of mycobacteriophage genomes [18], [30], [31], and the simplicity of phage isolation prompted us to isolate and characterize phages with the aim of studying their molecular biology and evolution, as well as constructing new tools for mycobacterial genetic analysis. In this report we describe the isolation and characterization of 18 mycobacteriophages, and the subsequent genomic sequencing and analysis of 12 of them in which novel features were observed.

## Materials and Methods

### Strains, Culture Media and Growth Conditions

*Mycobacterium smegmatis* mc^2^155, *M. bovis* var BCG strain Pasteur and *M. tuberculosis* H37Rv are lab stocks. The remaining mycobacterial species (*Mycobacterium kansasii* and *Mycobacterium fortuitum*) used throughout this study are clinical isolates, thus at least two of those clinical isolates were tested in our assays to avoid strain to strain variation which is mostly due to cell envelope changes. *M. smegmatis* mc^2^155 was routinely grown at 30°C or 37°C in Middlebrook 7H9 broth supplemented with 0.5% (v/v) glycerol, 10% ADS (albumin-dextrose-NaCl) and 0.2% (w/v) Tween 80 (hereafter designated as 7H9-ADS-Gly-Tw for short) as liquid culture medium, or in the same medium devoid of Tween 80 and supplemented with agar 1.5% (w/v) as solid medium (7H9-Gly agar). Growth of other mycobacterial species in liquid medium was performed on 7H9-ADS-Gly broth except for the amount of Tween 80 (0.05% (w/v) in the case of *M. tuberculosis* H37Rv and *M. bovis* BCG var. Pasteur; 0.5%–0.8% (w/v) for *M. kansasii* and *M. fortuitum*). Ca_2_Cl 2 mM was added to all the media used when mycobacteriophages were present unless otherwise indicated.

Liquid cultures were spun down at low speed at room temperature and washed twice with PhEB (Phage Extraction buffer, 50 mM Tris-HCl pH 7.6, 150 mM NaCl, 2 mM Ca^2+^, 10 mM Mg^2+^) to remove Tween, a known inhibitor of phage adsorption in mycobacteria [8]. When needed, indicator plates (IP) were prepared by mixing 10^7^ CFU of fresh *M. smegmatis* cultures with 4 ml of top agar (0.6% w/v agar in Midddlebrook 7H9 broth supplemented with 0.5% (v/v) glycerol and 2 mM CaCl_2_ unless otherwise indicated) and pouring the mix on top of 7H9-Gly agar plates supplemented with 2 mM CaCl_2_, hereafter mentioned as 7H9-Gly-Ca.

### Phage Isolation, Propagation and Titration of Lysates

Mycobacteriophages were isolated from environmental soil and water samples obtained from several places in Argentina. For this purpose samples were extracted by mixing with PhEB under gentle mechanical shaking at room temperature; the samples were centrifuged at low speed and the supernatant was filtered sterilized. The presence of phages in those filtered samples was detected by adsorption of 100–500 µl of filtrate to 100 µl of late log fresh culture of *M. smegmatis* mc^2^155 (grown at either 30°C or 37°C) for 30 min at room temperature, followed by addition of 4 ml of molten warm top agar, gentle mixing and finally poured on top of duplicate 7H9-Gly-Ca agar plates. After incubation for 24–48 h at 30°C or 37°C, plaque size, morphology and turbid/clear appearance were scored, single lysis plaques were picked and phages released with PhEB and further amplified as described above. Again a single plaque was picked and amplified to obtain a stock lysate which was kept at 4°C.

When necessary, phages were purified by banding in a CsCl density gradient. For this purpose, large scale plate lysates were prepared, clarified by low speed centrifugation, concentrated by precipitation with PEG 8000 and purified at 100,000 g; the band containing the phages was withdrawn and dialyzed against PhEB.

Genome sequencing of 1.5 µg of each phage DNA was performed at INDEAR, Argentina, by “whole genome shotgun sequencing” using a Life Sciences GS-FLX 454 sequencer with a redundancy of at least 10x. Genome sequence was obtained as a single contig for twelve of the phages, while six genomes were obtained as a two contigs or a small number of contigs. Bioinformatic analysis included sequence comparison by a set of programs including Gepard, genome annotation of open reading frames of selected phages was performed by using the DNAMaster program which includes GeneMark, Glimmer, Aragorn and tRNA Scan SE (available at http://cobamide2.bio.pitt.edu), while probable function assignment was done with HHPred and PFAM. Alignment and phylogenetic studies were performed by HMMER, ClustalW and MEGA5. Also tandem repeats were analized by Tandem Repeat Finder program. The sequence of the ends of the genomes of bacteriophages First, 20ES, 32HC, CRB1, 40AC, Jolie2 and 41HC were determined by PCR and sequencing of the product with primers listed in Table S1.

### Phage Characterization

Phage morphology and size was determined by Transmission Electron Microscopy (TEM) at two different facilities using different electron microscopes. A suspension of virions was applied to a sample grid with a carbon-coated nitrocellulose film, stained with either 2% uranyl acetate (w/v) or 2% phosphotungstenic acid (w/v), and examined in either a Jeol 1200 EX II TEM or a TEM Jeol 100CXII at a voltage of 85 KV; the magnification was of 50,000x and 140,000x.

### Pulse Field Gel Electrophoresis of Phage DNA

Phage genomic DNA was extracted from aliquots of phages embedded in agarose gel plugs from a stock lysate using a published protocol with minor modifications [17]. Briefly, aliquots of the lysates (equivalent to 10^9^–10^10^ PFU) were mixed with 1.5% agarose (w/v) and casted to form plugs. Once hardened the agarose plugs were incubated at 56°C for 12 h in a lysis solution (1% (w/v) N-lauryl sarcosine, 0.2% (w/v) SDS in 50 mM Tris pH 8.0–50 mM EDTA) in the presence of proteinase K (Fermentas, 0.5 mg/ml). Cell debris and any excess of proteinase K were removed by washing the plugs three times with washing solution (20 mM Tris.HCl; 50 mM EDTA at pH 8.0); and then resuspended in TE buffer. When needed, the plugs were heated at 80°C for 10 minutes and quickly cooled down on ice.

PFGE was performed using a CHEF DR II system (Bio-Rad, CA, USA) with a 1% (w/v) agarose gel in Tris-Borate electrophoresis buffer. Running conditions were set at 6 V/cm for 18 h with 2 seconds initial switch time and 10 seconds final switch time. After completion, gels were stained with ethidium bromide (10 µg/ml) and photographed under UV light. Lambda ladder PFGE Marker (New England Biolabs) was used as the reference marker.

### Effect of Temperature, Anti-mycobacterial Drugs and Cations in Mycobacteriophage Propagation

The effect of the temperature in the propagation of the isolated mycobacteriophages was evaluated by gently mixing ≈ 500 PFU with 1×10^8^ CFU of the indicator strain grown at either 30°C or 37°C, adding the mix to 4 ml of top agar and plating it on 7H9-Gly-Ca agar plates. Incubation of the plates for 48 h at 30, 37 and 42°C, was carried out with visual inspection every 12 h.

To assess the impact of the addition of sub-inhibitory concentrations of drugs, 7H9-Gly-Ca plates were supplemented with isoniazid (INH), ampicillin (Ap), or ampicillin sulbactam (Ap-Sb), (1 µg/mL, 5 µg/mL and 5 µg/mL respectively) that is, 1/5 of the Minimum Inhibitory Concentration of each antibiotic for the *M. smegmatis* mc^2^ 155 indicator strain. Plates were afterwards incubated at 30, 37 and 42°C, and plaque size visually scored every 12 h with an end point of 48 h.

The cation requirement for mycobacteriophage propagation was determined by plating 10 µl aliquots containing 1/10 dilutions (in 50 mM Tris.HCl pH 7.6–150 mM NaCl) of the mycobacteriophage under assay on base plates and top agar (in both cases devoid of CaCl_2_ supplement) containing either no cations, 10 mM CaCl_2_, 10 mM Mg Cl_2_ or 10 mM CaCl_2_/MgCl_2._ Plates were incubated at either at 30 or 37°C and visually inspected every 12 h for 48 h.

### Determination of Host Range

The capacity of the isolated mycobacteriophages to infect *M. fortuitum*, *M. kansasii, M. bovis* var BCG and *M. tuberculosis* H37Rv was tested by using as indicator strains 500 µL aliquots of fresh late log phase cultures of each species grown in 7H9-ADS-Gly-Tw (0.05–0.8% (v/v) Tween 80 depending on the species tested) and washed twice with medium devoid of Tween 80; two 5 µL aliquots containing 10^3^ y 10^7^ PFU of each mycobacteriophage were spotted on the indicator plates containing 2 or 10 mM CaCl_2_, the same concentrations of MgCl_2_ or a mixture of both. Plates were incubated at 30°C or 37°C for 3 days for *M. fortuitum* and *M.kansasii*. The infection of *M. tuberculosis* and *M. bovis* var BCG was done in two steps after the adsorption at room temperature for 30 min: an initial incubation for 3 days at 30°C or 37°C (depending on the infecting phage), followed by incubation at 37°C with daily observations.

### Isolation of *M.smegmatis* and *M. tuberculosis* Lysogens

Temperate phages usually produce plaques of turbid appearance, although in the case of mycobacteriophages, their turbidity may be slight. Thus, in order to detect temperate phages in our set, and to isolate *M. smegmatis* lysogens, we tested all the phages as if they produced turbid plaques. To this end, aliquots of *M. smegmatis* cultures were adsorbed at room temperature for 30 min with 100–200 PFU, mixed with 4 ml of top agar and poured on top of 7H9-Gly-Ca agar plates. Incubation of the plates was carried on at 30°C or 37°C (depending on the phage used) for three to four days with daily visual inspection. Colonies arising within each plaque of lysis were picked and separately streaked on 7H9-ADS-Gly agar plates several times. An average of 100 colonies surviving from each phage infection was afterwards tested for their lysogenic status and classified as wild-type, lysogens or phage-resistant mutants. This was accomplished by placing 50 µl drops containing ≈ 10^5^ PFU in a parallel pattern on the plate, tilting the plate and then letting it dry, followed by cross-streaking with a 10 µl aliquot from cultures of each putative clone on a plate, and recording the presence or absence of lysis upon incubation at 30°C or 37°C for 24–36 h. Cultures that did not show lysis, were further separated into lysogenic clones and phage resistant clones by triggering the prophage induction by UV treatment (30–60 sec) of 10 µl aliquots that were afterwards spotted on top of indicator plates (IP). Clones were further streaked on fresh plates for further characterization.

The search for *M. tuberculosis* lysogenic for the phages isolated was done using a comparable protocol: once the possible lysogens were picked from within lysis plaques, they were purified by streaking several times on fresh solid medium and incubated for 30 days at 37°C. Clones (10 from each infection) were patched on solid medium, grown at 37°C for 7 days and irradiated 60 seconds under UV light. At that point, molten top agar containing indicator *M. smegmatis* cells was carefully added. After hardening, plates were incubated overnight at 37°C and lysis detected visually.

## Results

### Isolation and Characterization of Mycobacteriophages

Our search for novel mycobacteriophages produced 18 of them over the last five years, isolated from over 100 environmental samples obtained at various geographical sites in Argentina. Single plaques were cloned and amplified; a first glance grouping by plaque morphology, showed nine phages that produced clear plaques (designated Hosp, Jolie1, Bahia1, Bahia2, First, 21AM, 21AS, Mine and 32HC) while 9 phages gave turbid plaques (designated 19ES, 20ES, 39HC, 40AC, 40BC, 41HC, Jolie2, CRB1, and CRB2).

Interestingly some of our phages produced tiny plaques (≈1–2 mm in diameter) that were hard to detect and manipulate and therefore made difficult the evaluation of their temperate or lytic nature. Phages may differ in host range, therefore, the very small plaques seen on isolation could be due to the utilization of a non optimal mycobacterial species as indicator bacteria. In support of that notion, plaque morphology changed upon propagation for phages Jolie1, Bahia1 and 32HC in *M. smegmatis* mc^2^155 suggesting that this strain may not be the natural host for those phages; i.e., Jolie1 (isolated at 30°C) initially displayed turbid plaques, 4 mm in diameter at that temperature, that switched to smaller (2 mm in diameter) and less turbid plaques upon repeated propagation at the same temperature. The observed change of plaque morphology of phage Bahia1 (isolated at 30°C) was more drastic, switching from 4 mm clear plaques to larger plaques (6 mm) with clear or turbid centers at the same temperature. Phage 32HC -isolated and handled at 37°C- also displayed plaque size reduction from 6 mm to 2 mm in diameter.

**Figure 1:**
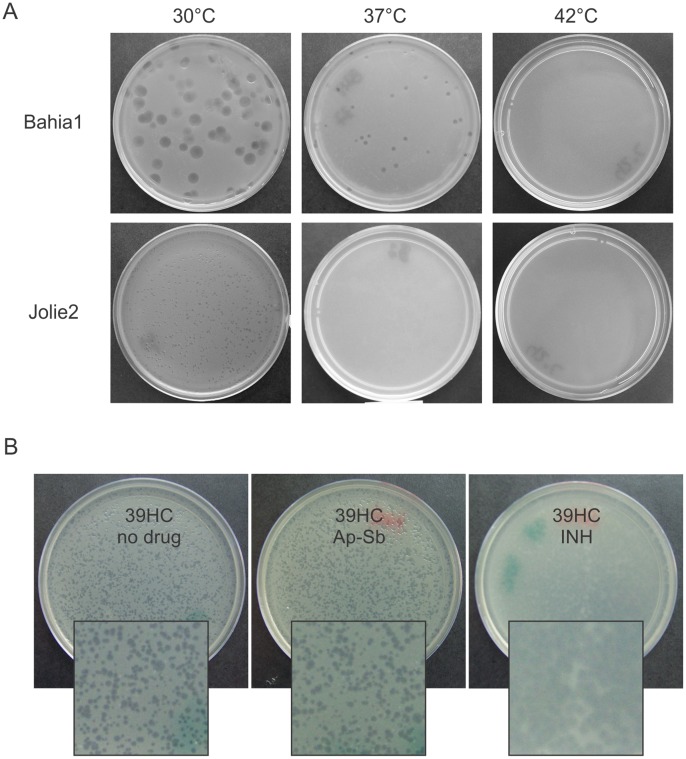
Plaque morphology changes by temperature or addition of antibiotics. **A.** Aliquots containing ≈100 PFU of phages Jolie1 and Bahia1 were adsorbed to aliquots of stationary *M. smegmatis* cultures grown at 30°C or 37°C, mixed with 4 ml of top agar and poured on 7H9-Gly-agar plates. Incubation proceeded for 48 h a30°C, 37°C or 42°C. For the sake of simplicity only the results at 30°C are displayed as results with cells grown at 37°C were identical. **B.** Bacteriophage 39HC was plated on lawns of *M. smegmatis* mc^2^155 grown on 7H9-Gly medium or in the same medium containing either Isoniazid (INH) or a commercial combination of Ampicillin-Sulbactam (Ap-Sb). Antibiotics were added to the bottom agar to final concentrations of 1.0 µg/ml (INH), or 5.0 µg/ml (Ap-Sb), respectively. For plate preparation, 4 ml of the top agar and 100 ml of an overnight bacterial culture (1×10^9^ CFU) were used. Magnification is shown at 2.5X of the original size.

Surprisingly, while all the mycobacteriophages gave lysis plaques at 30°C, ten of them (First, 20ES, 21AM, 21AS, 32HC, Bahia1, 40AC, 41HC, 19ES and CRB1) were able to form plaques (comparable to or smaller than the ones obtained at 30°C) at 37°C but none propagated at 42°C (Fig. 1A).

### Use of Sub-inhibitory Concentrations of Isoniazid or Ampicillin-sulbactam Allows for Detection of Small Plaque Forming Mycobacteriophages

The detection of mycobacteriophages giving very small plaques only at 30°C but incapable of forming detectable plaques at higher temperatures (such as Jolie2, 39HC and Bahia2), indicated that we may be overlooking a large fraction of the mycobacteriophage diversity present in our samples under the usual temperature screening conditions. Since we tested the influence of temperature in this phenotype by using the indicator strain grown at either 30°C or 37°C with negative results, we discarded changes in the cell envelope composition as the cause for the failure in propagation at the higher temperature. Thus, based on a previous report [32], we sought to improve their detection by using sub-inhibitory amounts of INH, a mycobactericidal drug that affects mycolic acid synthesis, thus damaging the cell envelope integrity. As shown in Fig. 1B for mycobacteriophage 39HC, plating of soil samples or already isolated small plaque forming phages on plates containing INH (1 µg/ml, MIC for *M. smegmatis* mc^2^155 = 5 µg/ml) led to a substantial increase in plaque size and turbidity and therefore, an improvement of visual detection and plaque cloning. Although this technique was successfully used at 30°C, it did not yield plaques at 37°C and 42°C in the phage sub-set that did not show plaques in the absence of drug, suggesting that previous failure to detect phage propagation at these temperatures was not due to poor phage release from infected cells.

The utilization of sub-inhibitory concentrations of Ap (5 µg/ml) combined with Sb -but not of Ap alone- also allowed for an increase in the size of the plaques, reinforcing the idea that a minor effect on cell wall integrity would help the release of the progeny phages, thus making plaques easier to spot, enumerate and manipulate.

### Morphometric and Genomic Analysis of the Isolated Mycobacteriophages

In order to characterize in depth our set of mycobacteriophages we studied their morphometric features by Transmission Electron Microscopy (TEM). The results showed that a majority of the phages have a total length of 190–350 nm with a few of them (phages CRB2, Mine and 40BC) in the 400–450 nm range (Fig. S1). As expected, and in agreement with previous publications, all the new mycobacteriophages described here displayed a morphology corresponding to the predominant *Siphoviridae* family [17], [33].

**Figure 2:**
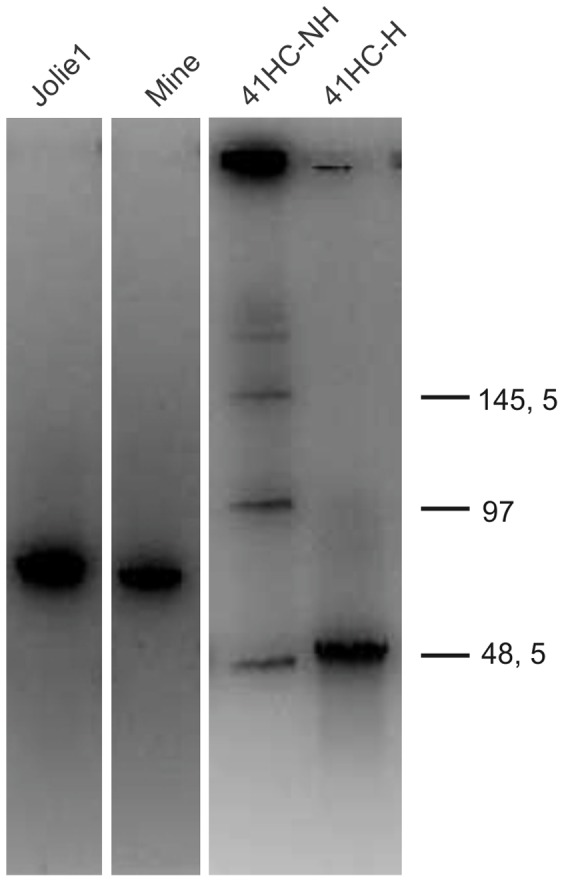
PFGE of DNA of mycobacteriophages Jolie, Mine, and 41HC. DNA of mycobacteriophages Jolie, Mine, and 41HC (not heated (NH) or heated (H) at 80°C for 10 min and cooled down in ice immediately prior to loading. DNA molecular weight (kb) on the standards is indicated at the right.

Preliminary results of the analysis of phage DNA by six restriction enzyme digestion showed that the phages were clearly different although some shared bands of comparable size for some of the restriction enzymes used (data not shown). The genome size of these newly isolated mycobacteriophages was determined by Pulse Field Gel Electrophoresis (PFGE); the results obtained showed that most of them (12/18 phages) had genomes of 42–55 kbp, while six showed genome sizes in the range of 62–70 kbp. Interestingly several of the mycobacteriophages analyzed showed bands compatible with multimers of their genomes, requiring a heating step prior to loading into the gel to avoid this behaviour (Fig. 2).This result suggested the presence of cohesive ends [34] in our mycobacteriophages genomes, in good agreement with an almost 70% of mycobacteriophage genomes with such features previously described (http://phagesdb.org/).

Further characterization was accomplished by genomic sequencing of the 18 mycobacteriophages, of which twelve were obtained as single contigs. Comparison of the sequence similarity of the twelve phages in a dotplot matrix allowed to form four groups, indicating that phages Mine, CRB1, First, 20ES, 40AC, 41HC were related, while phages 40BC, Hosp, Jolie1, 39HC also displayed sequences similarities of more than 50% between them and thus were grouped together. When compared to already described phages, we were able to include our phages in the mycobacteriophage clusters described by Hatfull’s group [35] REF. Most of our phages belonged into Group A, four of them fell into Group B with only one phage (Jolie2) belonging into Group G; however, one phage (32HC) remained as a new singleton through all the bioinformatic analysis (Table 1).

**Table 1:** Genometrics of the novel mycobacteriophages isolated.

Phage	Size(bp)	GC%	No. ORFs	tRNA	Ends	Sequence of ends (5′–3′)	Cluster
First	53028	63.42	95	1	10 pb 3′	CGGTCGGTTA	A
41HC	52943	63.34	99	1	10 pb 3′	CGGTCGGTTA	A
40AC	53396	63.34	95	0	10 pb 3′	CGGTCGGTTA	A
20ES	53114	63.43	96	1	10 pb 3′	CGGTCGGTTA	A
CRB1	52953	63.23	97	1	10 pb 3′	CGGTCGGTTA	A
Mine	71591	69.36	99	0	Circ Per		A
32HC	50768	65.73	91	0	13 pb 3′	TCCGATGCGGTGA	singleton
Hosp	70667	69.89	101	0	Circ Per		B
Jolie1	71058	69.88	99	0	Circ Per		B
39HC	71565	70	103	0	Circ Per		B
40BC	71565	70	103	0	Circ Per		B
Jolie2	44296	68	62	0	10 pb 3′	CCCGTGGCAT	G

The study of the linear genomes of these phages showed that their ends were either circularly permuted or cohesive. After determination of the sequence by PCR, of the latter, we found that five of them had a 10 bp 5′-CGGTCGGTTA-3′sequence, matching one of the sequences described for other mycobacteriophages also belonging to Cluster A. Mycobacteriophage Jolie2, contained a different 10 bp 5′- CCCGTGGCAT-3′ that linked it to Cluster G mycobacteriophages. One phage (32HC) contained a 13 bp 5′-TCCGATGCGGTGA-3′sequence that has not been previously reported, indicating again that this phage is a singleton. The remaining mycobacteriophages, contained circularly permuted ends and belonged into Cluster B, all which members contained this type of end. However, an interesting case is represented by mycobacteriophage Mine, that shows circularly permuted ends despite of grouping with Cluster A phages (Table 1).

### Cation Requirements and Host Range of the Novel Mycobacteriophages

Early work by Hatfull’s group showed that a strict dependence of the presence of Ca^2+^ ions (≥1 mM in both bottom and top agar) for a productive infection of slow-growing mycobacteria by the temperate phage L5 [36]. Surprisingly, D29, a mycobacteriophage that is a close relative of L5, did not show that requirement, being able to propagate although at a reduced efficiency (two– to three-fold) in both slow and fast growing mycobacteria [36]. Since one of the aims for this work was to establish the host range for our mycobacteriophages, we initially determined the nature and concentration of the cation(s) that allowed for optimal propagation in *M. smegmatis* mc^2^ 155. For this purpose we tested the effect of 10 mM CaCl_2_, 10 mM MgSO_4_ and a 10 mM mix of both CaCl_2_ and MgSO_4_ on phage propagation. The results determined that most of the phages required the presence of a divalent cation without a preference for either Mg^2+^ or Ca^2+^ in the medium; as opposite to phages 20ES, 21AM, 40AC, 41HC and CRB1 that were highly dependent on the presence of Ca^2+^ (Table S2). Surprisingly, phage 19ES was able to infect and propagate in the indicator strain with the same ability regardless of the presence or absence of added cations (Fig. S2). Thus, 19ES shares with TM4 the characteristic of not requiring the addition of extra divalent cations for its adsorption, suggesting a different mechanism and/or receptor(s) for infection.

With the goal of identifying amongst these newly isolated mycobacteriophages which one(s) were able to infect *M. tuberculosis, M. bovis* var BCG and NTM, we tested different cation(s) concentrations and at two titres (low, 10^7^ PFU/spot; high, 10^3^ PFU/spot) of phages on *M. tuberculosis*, *M. bovis* var BCG, *M. fortuitum,* and *M. kansasii*. None of the phages were able to infect *M. fortuitum* at low titres, but phages First, 20ES, 21AM, 21AS, 40AC, 41HC, CRB1, Bahia1, Bahia2 and CRB2 showed faint haloes when 10^7^ phage particles were used. The same results were obtained for *M. kansasii* except that only phages First, 20ES, 21AM, 21AS and CRB1 displayed faint haloes at the low phage dilution used while the remaining phages did not give any visible alteration of the cell lawn. Similar results were reported by Rybniker *et al.* for the host range of several well-known mycobacteriophages on a larger set of mycobacterial species [37].

Interestingly, four mycobacteriophages (First, 20ES, 21AM and 21AS) were able to propagate in *M. tuberculosis* H37Rv and *M. bovis* var BCG at the lowest titre tested. In those cases there was a considerable effect of the cation and concentration used, with 10 mM CaCl_2_/MgCl_2_ giving very large plaques (Fig. S3). There was no difference in the frequency of plaque formation between *M. smegmatis* and *M. tuberculosis*, suggesting that these phages had the same ability to propagate in both species (data not shown).

#### A sub-set of phages lysogenize *Mycobacterium smegmatis* and *Mycobacterium tuberculosis*

Mycobacteriophages have been a highly valuable resource for the development of mycobacterial genetics, taking part as scaffold for transposon delivery systems and providing the genetic means for the design of integrative plasmid vectors [21]. The importance of such tools led us to analyze the ability of our mycobacteriophages to lysogenize *M. smegmatis* and *M. tuberculosis*. To this end, we isolated *M. smegmatis* clones growing within lysis plaques generated upon phage infection. From those colonies, the three expected types (lysogens, non-lysogenic clones and phage resistant clones) were obtained. Clones belonging to each class were identified by either susceptibility or resistance to the phage used for the infection or by the ability to release phages upon exposure to UV-light. In this way, we identified eight phages (20ES, First, 21AM, 21AS, 32HC, 19ES, 41HC and CRB1) that were able to lysogenize *M. smegmatis*. Of those, four (First, 20ES, 21AM and 21AS) that were able to infect *M. tuberculosis*, displayed a temperate behaviour, as we could detect lysis upon UV treatment of putative lysogenic *M. tuberculosis* cells growing inside lysis plaques. Thus, the described mycobacteriophages were initially considered logical candidates for bioinformatic analysis aiming to their use in the construction of novel integrative vectors. A summary of the information of our set of mycobacteriophages is described in Table S3.

#### Bioinformatic analysis of the putative temperate mycobacteriophage First genome

**Figure 3:**
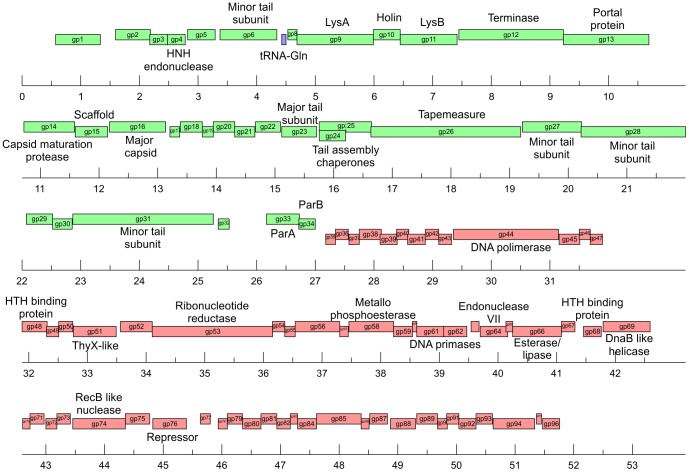
Genome organization of mycobacteriophage First. The scheme shows the gene annotation of mycobacteriophage First, the dashed arrow at gp25 represents the (−1) frameshift that generates gp 24 and gp25.

The fact that First was capable of infecting both *M. smegmatis* and *M. tuberculosis*, and its initial classification as temperate led us to analyze its genome to detect the presence of putative *att*P, *int* and *xis* sequences. Bioinformatic analysis allowed for the identification of open reading frames and possible functions to be assigned (Table S4). The sequence of the ends of this 53028 bp mycobacteriophage, determined by PCR and sequencing of the product, showed a 10 bp 3′ defined overhang with the sequence 5′-CGGTCGGTTA- 3′. The genomic organization of First (represented in Fig. 3) is highly similar to the one displayed by members of Group A, having the gene order terminase, portal, protease, scaffold, capsid, head-tail joining genes, major tail subunits, tapemeasure protein and minor tail protein subunits. First also shares another feature of Cluster A mycobacteriophages in having additional non-structural genes between the terminase and the left end of its genome, identified as the lysis cassette genes. We also detected the presence of a translational (−1) frameshift located between First ORF24 and ORF25, encoding for the tail assembly chaperones; other common feature of Cluster A members. As many other mycobacteriophages, First encodes proteins hypothetically involved in metabolism of nucleotides, such as thymidylate synthase and ribonucleotide reductase. The sequence and annotation of First has been deposited in GenBank under accession number JX899358.

During our preliminary characterization, First behaved as a temperate phage, producing *M. smegmatis* clones that were resistant to it but produced high titer lysates upon UV induction. Thus, those putative First lysogens were an initial proof that this phage may be temperate and therefore of potential use in developing integrative vectors. Genomic analysis of Cluster A mycobacteriophages showed that integration cassettes are close to the center of their genomes in spite of genome length differences. However, strikingly, First does not contain any identifiable integrase/excisionase genes and/or the corresponding attachment site, *att*P, in spite of careful search with several bioinformatic tools as well as manual analysis of the sequence. In its place genes with homology to *par*A and *par*B genes were detected.

The presence of these genes led us to search for putative repeated tandem sequences possibly functioning as centromeres [38]. We were able to detect several sequences by bioinformatic analysis bearing the characteristic tandem repeats in different positions of the phage genome; based on reports indicating that usually the sequence acting as centromere is located adjacent *par*A, we tentatively postulated the sequence 5′-GAGTCCTCGAGGAGTCGAATAGTC- 3′ (repeated twice) at nucleotide positions 26102–26159 as the possible centromere for this phage, although functional corroboration is needed.

#### Presence of *par*A or *par*A/*par*B genes is widespread in heritable mycobacteriophages

**Figure 4:**
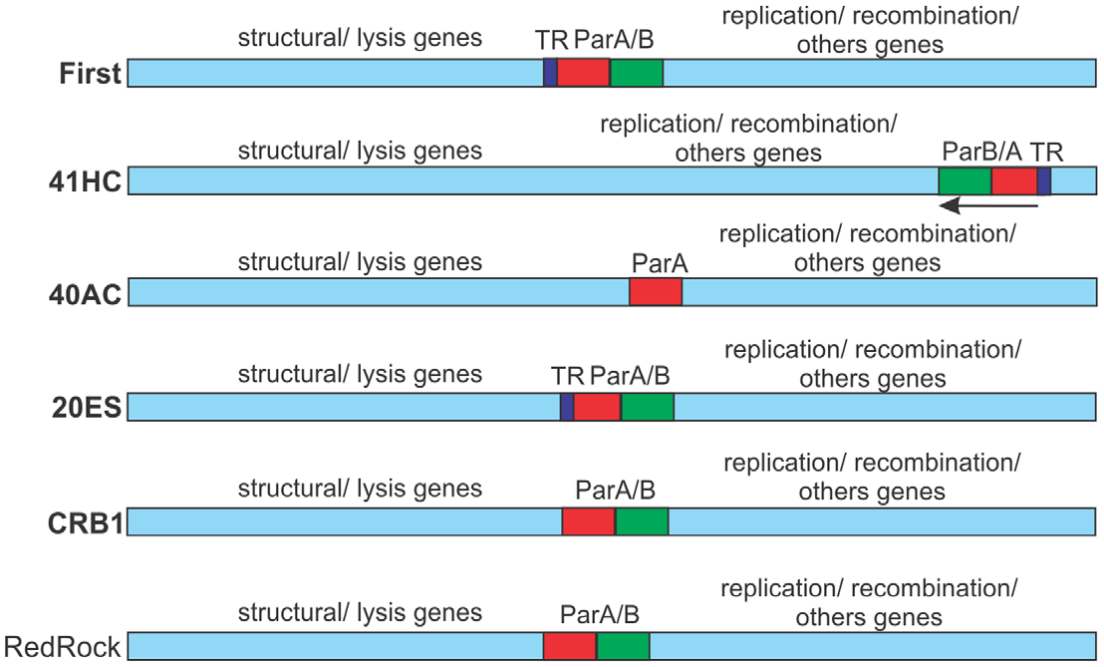
Partitioning loci location in mycobacteriophages. Schematic representations of the mycobacteriophage genomes showing the location of putative *par*A and *par*B genes and sequences of tandem repeats (TR) functioning as putative centromers. Genes transcribed leftward are indicated. RedRock is included as a representative of *par*A/*par*B containing mycobacteriophages as published by Hatfull [31].

Due to the detection of *par*A and *par*B genes in First, and taking in consideration previous reports showing the presence of such genes in *Leptospira* [39], [40] and *Escherichia coli* [41], [42] bacteriophages and in some mycobacteriophages [31], we analyzed the presence of the mentioned genes in all our isolates as well as their relatedness to others available in public databases. The results showed that *par*A could be identified in mycobacteriophages 20ES, 40AC, 41HC, CRB1 in addition to First. Also, analysis of the partial genome sequences of mycobacteriophages 21AM and 21AS, revealed the presence of hypotetical ParA proteins. Interestingly, *par*B genes were found in all the above mentioned mycobacteriophages excepted for phage 40AC, that behaves as a lytic phage. Thus, from our reported eight “lysogenic” mycobacteriophages, six contain *par*A/*par*B genes ((Table 2). Moreover, a search in a non-redundant database showed the presence of centrally located *par*A and *par*B genes in the genomes of Cluster A mycobacteriophages RedRock, PackMan, Alma, EricB, Hammer, Gladiator, Jeffabunny, DaVinci and Blue7 and in mycobacteriophage TA17A, a Cluster B member [35], [43]. Our own phages also display a central position for *par*A and *par*B genes except in the case of phage 41HC, in which the partitioning locus is located in a terminal position (Fig. 4).

**Table 2:** Inheritance of “temperate” mycobacteriophages.

Mycophage	ParA/ParBcoordinates	tandem repeats spanning region^a^	Integrase	*att*P	Repressor^§^
First	26162–26993	26102–26159	N/A	N/A	+
41HC	46559–47390	47399–47444	N/A	N/A	+
20ES	26076–26925	26020–26062	N/A	N/A	+
CRB1	26655–27597		N/A	N/A	+
32HC	N/A*		Tyr	Msmeg_5758	ND^b^
21AM^#^			N/A	N/A	ND^b^
21AS^#^			N/A	N/A	ND^b^

(#)- Genome sequence not assembled, so no accurate position for *par*A and *par*B genes, tandem repeat sequences (TRs) are listed for 21AM and 21AS mycobacteriophages.(*) N/A- Not applicable; (a)-TRs upstream *par*A gene are possible centromeres, thus only TRs found at this position are reported; (b)- Not detected bioinformatically; (^§^)- Bioinformatically detected.

**Figure 5:**
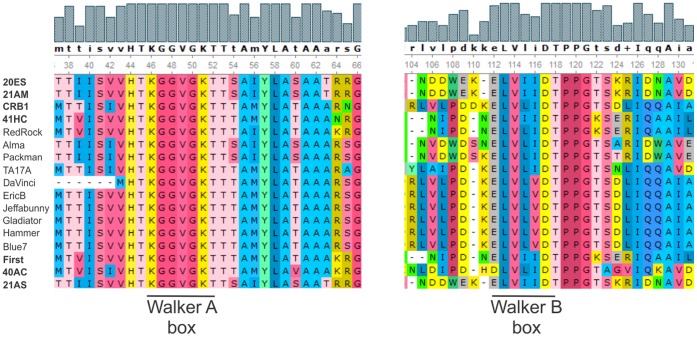
Alignment of the Walker A and B motifs in ParA proteins.

**Figure 6:**
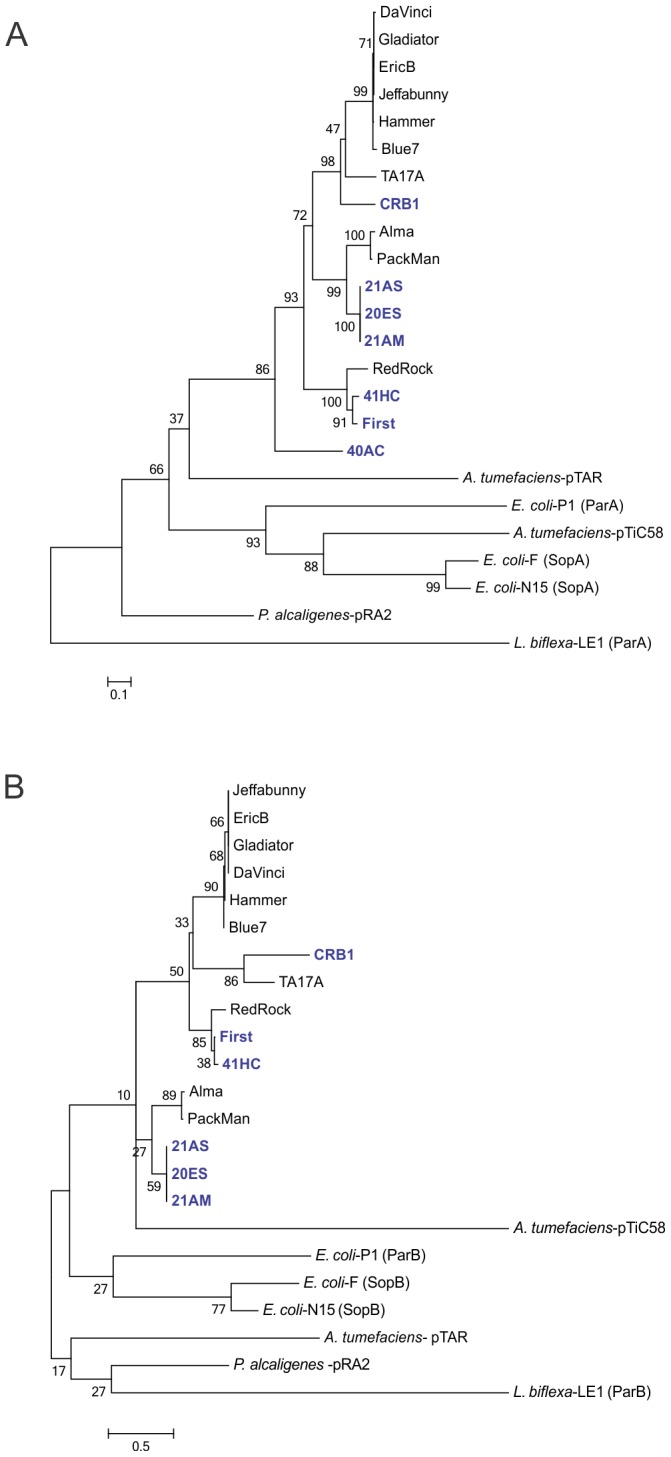
Phylogenetic analysis of mycobacteriophage partition proteins present in plasmids (pTAR, pTiC58, F, pRA2), non-mycobacterial bacteriophages (P1, N15, LE1) and mycobacteriophages. The neighbourjoining (NJ) tree was obtained with the MEGA5 software using an output from an alignment in ClustalW; bootstrap values from 1000 iterations are shown for ParA (A) and ParB (B) proteins.

Our identification of several *par*A genes prompted the analysis of their phylogenetic link; the results located the Walker A and B boxes, necessary for ParA function, that are highly conserved in this family of proteins [38], [44] (Fig. 5). Interestingly mycobacteriophage 40AC, which lacks a *par*B gene but contains a *par*A gene, does not group with the rest of the mycobacteriophages described, suggesting that it underwent a deletion and entered a lytic way of life at a distant evolutionary point (Fig. 6A).

We also analyzed the relatedness of ParB proteins in our set of mycobacteriophages. As we did not find a difference in the general linkage when other ParB proteins from plasmids and other bacteriophages were considered, we included the data for the different *par*B genes regardless of origin. Our results (Fig.6B) suggest that the linkage between ParB proteins correlates to the linkage of ParA proteins as their relative group position when compared to their most related neighbours, suggesting that evolution driven changes for ParA and ParB may be simultaneous. As an example of this, the ParA proteins of phages 21AS, 21AM and 20ES are clustered and linked to ParA proteins of phages Alma and Packman, and this association is maintained for their ParB proteins. The same result is observed when the relatedness of ParA and ParB of phages First, RedRock and 41HC is analyzed. In this case, although the group position relative to other groups changed, the three ParA and ParB proteins show the same linkage between them (Fig. 6B). These observations probably reflect the functional link between ParA and ParB proteins, however, functional analysis of this novel mechanism of inheritance of mycobacteriophage needs to be performed to obtain more information.

## Discussion

### Modification of the Isolation Protocol Reveals New Features of Mycobacteriophages

It can be stated with little doubt that mycobacterial genetics became tractable after the introduction of mycobacteriophage derived tools since these biological entities provided materials for the construction of integrative plasmid vectors, transposon delivery systems and very importantly, for the precise deletion of genes from the mycobacterial chromosome and for the introduction of point mutations in any given gene [10], [28], [45]. The largest and most comprehensive body of work in the mycobacteriophage field was produced by Hatfull’s group, who isolated more than 3,300 mycobacteriophages, sequencing and annotatting a large number of their genomes [43]. Inspired by the work mentioned above, we sought to isolate and characterize mycobacterial phages. We used *M. smegmatis* mc^2^155 as host strain throughout the search, detection and amplification of mycobacteriophages present in environmental samples collected at different points in Argentina. As expected, because of the bias on the host species, none of our phages showed ability to form plaques in NTM such as *M. fortuitum* or *M. kansasii* when used at low titers. Thus, it would be profitable to use NTM species in order to look for phages able to replicate in that group to take advantage of the bacteriophage-based tools that would be generated.

During our initial screening, by serendipity we isolated a mycobacteriophage capable of replication at 30°C but not at higher temperatures, thus we introduced several changes in the isolation protocol, broadening the search by using both 30°C and 37°C as incubation temperatures. In this way we isolated 18 mycobacteriophages, of which ten were able to form lysis plaques at both temperatures, while the remaining eight only propagated at the lower temperature. This intriguing feature is not due to a failure to adsorb to the host cell due to changes in temperature-driven mycobacterial cell envelope composition (perhaps affecting the structure of mycobacteriophage receptors) since phage propagation was observed when adsorption was performed only at 30°C regardless of the temperature at which the indicator strain was grown. Thus this is the first report of a mycobacteriophage isolated at 30°C and incapable of propagation at higher temperature. So far there is no explanation for this behaviour; given its possible use as a suicide delivery vector, more research on this topic is warranted.

We also characterized the cation dependence of our mycobacteriophages, finding interesting results; while several phages showed improved amplification in the presence of Ca^2+^ (phages 21AM, 20ES, 40AC, 41HC and CRB1), a second set demonstrated no preference for either Ca^2+^ or Mg^2+^ most likely reflecting an nonspecific improvement in virion stability. Surprisingly, one phage (19ES) was able to infect *M. smegmatis*, giving the same level of amplification regardless of the presence or absence of Ca^2+^ or Mg^2+^. Divalent ions are required for stability of mycobacteriophage virions or –in the case of Ca^2+^- the adsorption step [36], thus our results suggested that phage 19ES has either a very low calcium requirement, totally different receptors than the rest of phages characterized or an unusual mechanism of adsorption. Cation requirement for mycobacteriophage infection has been studied for very few mycobacteriophages such as L5 and D29 [36], it is highly possible that mycobacteriophages (such as TM4 and 19ES) that are not bound to those requirements may be present in the biosphere. This has importance not only on the understanding of molecular evolution of mycobacteriophages but also on studies of mycobacterial phage receptors. Nine of our phages yielded clear plaques (Hosp, Jolie1, Bahia1, Bahia2, First, 21AM, 21AS, Mine and 32HC) while the remaining nine phages gave turbid ones (19ES, 20ES, 39HC, 40AC, 40BC, 41HC, Jolie2, CRB1 and CRB2). In some cases we observed the same phenomena described by Morris *et al.*, who reported that phage Giles was able to form plaques that were not clear neither intensely turbid [46], its temperate nature was confirmed by detection of the release of virions from purified putative lysogens. Following identical criteria we tentatively considered all our mycobacteriophages as temperate unless an absolutely clear plaque was produced upon infection.

TEM analysis of our phages showed that all of them belonged into the Siphoviridae family, an expected result as the siphoviridal morphology is predominant in mycobacteriophages [17]. Likewise, morphometric data as well as genome size characterization by PFGE, gave results within the range of other previously characterized mycobacteriophages, (http::www.phagedb.org). However, we have not identified any mycobacteriophage with a large size genome (larger than 120 kbp), probably due to the relatively small number of our set of mycobacteriophages. Large genome size and transducing ability seems to correlate in mycobacteriophages as shown by phages I3 and Bxz1 [47], [48]. In agreement with those observations, none of our isolates (bearing genomes under 100 kbp) behaved as a transducing phage (Franceschelli, J. J., personal communication).

Genometrics of our mycobacteriophages are in good agreement with published data, as phages belonging to the same cluster have similar genome size, G+C content, and the same type of ends [31]. Surprisingly, mycobacteriophage Mine groups into Cluster A, but this particular phage shows circularly permuted ends instead of the cohesive ends typical of Cluster A mycobacteriophages, has a genome size and a G+C content more similar to phages belonging to cluster B and is unable to form plaques at 42°C as opposite to published Cluster A phages. We are currently annotating its genome in order to gain further knowledge on this peculiar phage.

There is a growing interest in bacteriophages as components for different biological purposes (phage display, biosensors, diagnostics, microbial genetics, etc.), for which isolation, visual detection and enumeration – limited by plaque size- are essential steps [49]. Three of our mycobacteriophages consistently produced very small pinpoint size plaques of lysis, thus we tested if a published simple method would increase their size, overcoming this difficulty [32]. The method is based in the addition of sub-inhibitory concentrations of drugs affecting cell envelope biosynthesis of the host cell, thus allowing an easier release of phage progeny. The use of INH -an inhibitor of mycolic acids, essential components of the mycobacterial cell envelope- or Ap-Sb -inhibitors of peptidoglycan synthesis and ß-lactamases respectively- allowed for the production of larger plaques, therefore making it easier to manipulate plaques and to pick up putative lysogens from within the plaque.

Thus, the use of this simple trick as well as the utilization of low incubation temperatures and the absence of divalent cations during the isolation procedure would most likely expand the detectable mycobacteriophage world and therefore, provide more information on their molecular biology and evolution.

### Mycobacteriophage Genomes Frequently Contain Partition Loci

In order to further characterize our mycobacteriophages we determined their temperate or lytic nature. Eight of the eighteen mycobacteriophages analyzed (20ES, First, 21AM, 21AS, 32HC, 19ES, 41HC and CRB1) behaved as temperate although only four of them (First, 41HC, 21AS and 32HC) produced stable lysogens at good frequency (11–45%) in *M. smegmatis* while the remaining phages showed less ability to lysogenize (≤5%). One of such cases was phage First that showed 45% of “lysogenization”. Thus, our results are in good agreement with those found for other mycobacteriophages such as Giles, Halo and Angel which display a frequency of lysogenization of 2–5% [19], [46], as well as for L5, which lysogenizes *M. smegmatis* at a frequency of 22% [50]. Lysates obtained by UV induction of four of the temperate phages (First, 20ES, 21AM and 21AS) amplified in *M. smegmatis* were capable of infecting and forming plaques in *M. tuberculosis* H37Rv at low titers. This is very different of what was reported by Sampson *et al.* who showed that phages BPs and Halo amplified in *M. smegmatis* initially displayed a diminished frequency of plating in *M. tuberculosis* (10^5^-fold reduction) [19]. However, amplification of a plaque obtained on the *M. tuberculosis* lawn was able to form plaques with the same efficiency in both *M. tuberculosis* and *M. smegmatis* suggesting that mutants of the original phages with an expanded host range were selected after the initial infection. Considering that *M. smegmatis* is the species regularly used for mycobacteriophage isolation and propagation (excepting TM4 which was isolated from a *M. avium* lysogen), and that only a small fraction of mycobacteriophages described so far infect *M. tuberculosis*, these phages were initially considered an useful finding since construction of integrative vectors based on them would also be of use for *M. tuberculosis* genetics [51].

However, bioinformatic analysis of mycobacteriophage First, our firstly chosen candidate for genetic analysis, showed the absence of genes encoding possible integrases/excisionases and of *att*P sites, suggesting another mechanism underlying its behaviour as “temperate”. A plausible explanation stems from the detection in its genome, of genes involved in the partition of genetic units such as plasmids [52], [53], chromosomes [54], [55] and -much less frequently- bacteriophages [39], [56]. Those genes have homology to *par*A and *par*B genes and display a genetic organization compatible to the one described for the type 1 b partitioning loci in which a centromere is located upstream of *par*A and *par*B [38]. Moreover, our *par*A -*par*B locus encodes for proteins of similar size to the ones encoded by members of the type 1 b partitioning loci as described by Gerdes [38]. This feature seems to be a quite common trait in mycobacteriophages that are “heritable” as several of our sequenced phage genomes revealed the presence of *par*A-*par*B loci. In only one case a phage (40AC) contained a *par*A but no *par*B gene, however this phage in particular behaved as lytic and probably reflects a deletion of the *par*B member of the locus. Interestingly, the partitioning loci are usually located in the middle of the genome of our mycobacteriophages (Fig.4). An exception for this central location is phage 41HC which contains a *par*A *par*B locus placed almost at the end of its genome. In some cases, we were able to detect one or more tandem repeats (TR), the *cis* acting sequences to which ParB proteins bind [38], [44]; some of those TR are positioned close to the *par*A-*par*B loci (Fig.4), reinforcing our hypothesis that they are true partition systems involved in the inheritance of mycobacteriophages; in spite of that, functional analysis is required to identify the functional TR.

Partitioning loci have been described more frequently on plasmids and bacterial chromosomes, however very few bacteriophages infecting *E.coli* (such as P1, P7, N15) have been found to contain a partitioning locus in spite of the large number of phages described for this genus and other gram negative bacteria [41], [42], [57], [58]. Interestingly, the only leptospiral phage isolated and characterized in detail also contains *par*A and *par*B genes [40], whether this is a regular feature or not will need the study of more leptospiral phages, now in progress in our laboratory. However, we demonstrate herein, that partitioning systems are a functional mechanism for inheritance of mycobacteriophages. Intriguingly, we found *par*A and *par*B genes in roughly 50% of our isolates, although they are typically less frequently described in the available databases containing hundreds of mycobacteriophages. The reason for their over representation in our set is not clear at the moment, however it raises questions on why this peculiar mechanism for inheritance is present in mycobacteriophages, avoiding the more commonly found mechanism of lysogenization. Considering the little enthusiasm that mycobacteria seem to display for genetic exchange, as judged by the very few indigenous plasmids and general transducing phages, it is tempting to postulate that mycobacteriophages have acquired partitioning loci as an alternate way to “travel along” with mycobacteria without being discarded too soon and too often and without ending integrated in the mycobacterial chromosome.

### Conclusion

We herein report the isolation and characterization of new mycobacteriophages; the most striking features observed arose from the use of different conditions for isolation, playing with temperature of infection, host strain used, cations added into the medium and improvement in the release of phage progeny by addition of sub-inhibitory concentrations of antibiotics.

Strikingly, several of our phages contain partitioning loci that are far less common in other bacteriophages such as coliphages; although they are present in other mycobacteriophages, this is the first report performing a phylogenetic analysis. Moreover, the proof that ParA/ParB proteins are functionally related to the phage “inheritance”, is given by one of our mycobacteriophages that lacks its *par*B cognate gene and displays a lytic phenotype. Our results suggest that a fraction of the mycobacteriophage population may have adopted a non-integrative mechanism for their inheritance in addition to the more common integration/excision system carried on by specialized enzymes. A plausible hypothesis is that, by avoiding integration, the pair-*Mycobacterium*/mycobacteriophage ensure themselves that “junk” DNA generated by random mutations impeding excision, will not be stably inherited. The evolutionary and practical advantages of this strategy for both, phage and bacteria, are still far from being understood but undoubtedly warrant future studies.

## Supporting Information

Figure S1 **Mycobacteriophage virion morphologies.** Transmission Electron Microscopy micrographs of representative particles of eighteen mycobacteriophages are shown. Bar corresponds to 100 nm
(TIF)

Figure S2 **Effect of the presence of cations on the propagation of mycobacteriophages.** Ten ml aliquots of serial dilutions of mycobacteriophages 19ES and Mine were spotted on 7H9-Gly-agar plates containing the *M. smegmatis* mc^2^ 155 indicator strain in top agar with the addition of either 10 mM Ca^2+^, 10 mM Mg^2+^ or their combination. Medium with no extra divalent salts was used as control. The cations were present in both bottom plate and top agar layer. PFU of each phage spotted for each condition are indicated on the left side.
(TIF)

Figure S3 **Four mycobacteriophages infect** ***M. tuberculosis*** **H37Rv.** Aliquots containing 10^3^ PFU of mycobacteriophages First, 20ES, 21AM and 21AS were spotted onto a lawn of *M. tuberculosis* H37Rv grown on 7H9ADS-Gly plates and incubated at 37°C for 10 days before visual inspection.
(TIF)

Table S1 **Oligonucleotides used to amplify the mycobacteriophage genomic ends.**
(DOCX)

Table S2 **Cation dependence of mycobacteriophage infection of** ***M. smegmatis*** **mc^2^ 155.** Phage titre assays were performed using Middlebrook 7H9-Gly agar. An aliquot of a culture of *M.smegmatis*, used as indicator strain, was included on a top agar made with the same medium. In all cases both medium were either devoid of calcium and magnesium salts or supplemented with 10 mM of each cation of their combination. The same conditions were used during the adsorption step. Aliquots containing 1/10 dilutions of each phage were spotted on the top agar. The minimum PFU required to produce detectable lysis was visually determined.
(DOCX)

Table S3 **Characteristics of novel mycobacteriophages.** (/) in the “cation requirement” column means no preferences for Ca^2+^ or Mg^2+^; Mtb, *M. tuberculosis* H37Rv; BCG, *M. bovis* var BCG strain Pasteur; Msmeg, *M. smegmatis* mc^2^155; D, sequence deposited in GenBank; AS, assembled sequence; PAS, partially assembled sequence.
(DOC)

Table S4 **Annotation of ORFs in the genome of mycobacteriophage First.** Coordinates and putative functions of First are indicated. A tRNA:tRNA-Gln(ctg) is encoded at coordinates 4425–4503 bp but not listed.Direction of transcription, F, forward (leftwards); R, reverse (rightwards).
(DOCX)
