# Supplementary figures and images for: Analysis of Novel Mycobacteriophages Indicates the Existence of Different Strategies for Phage Inheritance in Mycobacteria
Source: PLoS One. 2013 Feb 28;8(2):e56384. doi: 10.1371/journal.pone.0056384 (PMC3585329; doi:10.1371/journal.pone.0056384)

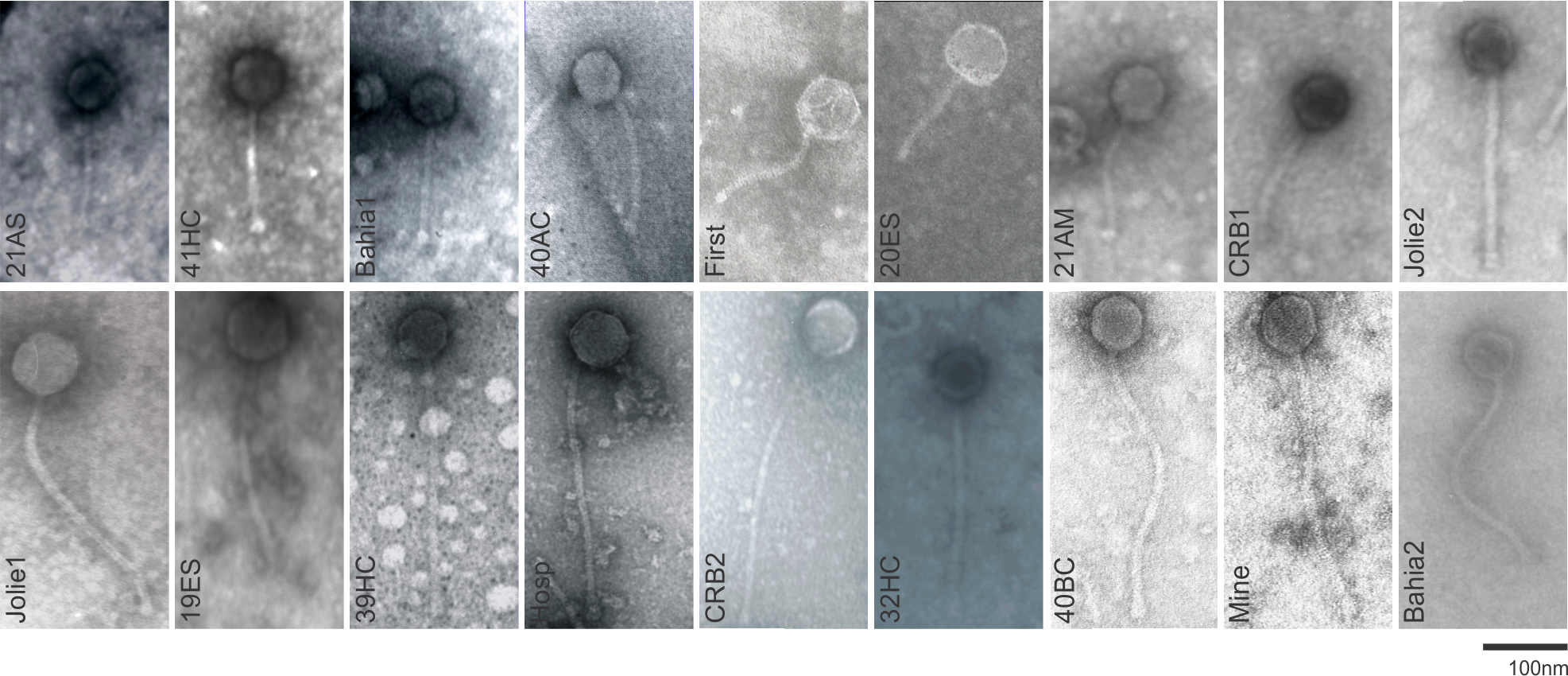

Supplement: Figure S1 — Mycobacteriophage virion morphologies. Transmission Electron Microscopy micrographs of representative particles of eighteen mycobacteriophages are shown. Bar corresponds to 100 nm (TIF) [file pone.0056384.s001.tif]

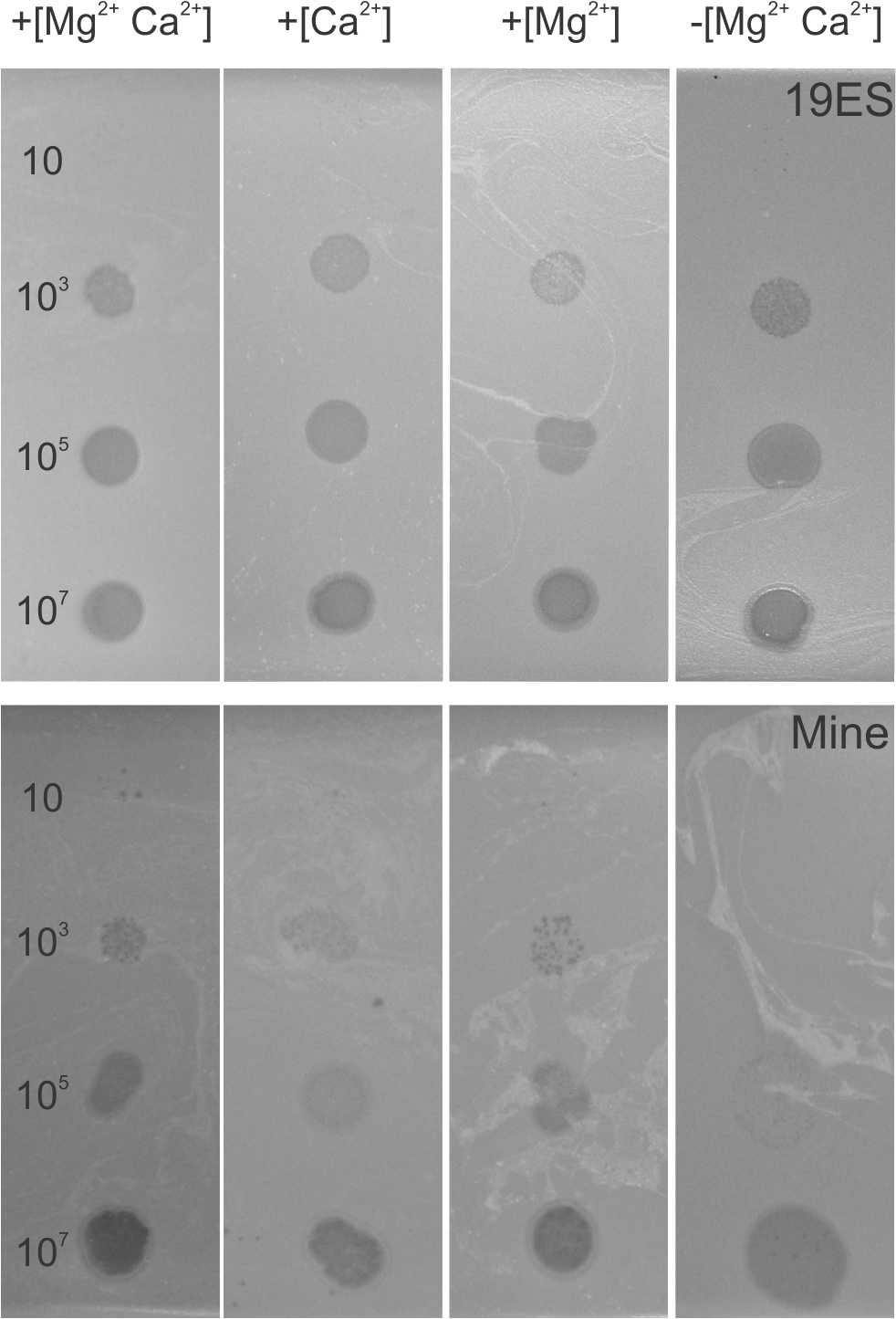

Supplement: Figure S2 — Effect of the presence of cations on the propagation of mycobacteriophages. Ten ml aliquots of serial dilutions of mycobacteriophages 19ES and Mine were spotted on 7H9-Gly-agar plates containing the M. smegmatis mc2 155 indicator strain in top agar with the addition of either 10 mM Ca2+, 10 mM Mg2+ or their combination. Medium with no extra divalent salts was used as control. The cations were present in both bottom plate and top agar layer. PFU of each phage spotted for each condition are indicated on the left side. (TIF) [file pone.0056384.s002.tif]

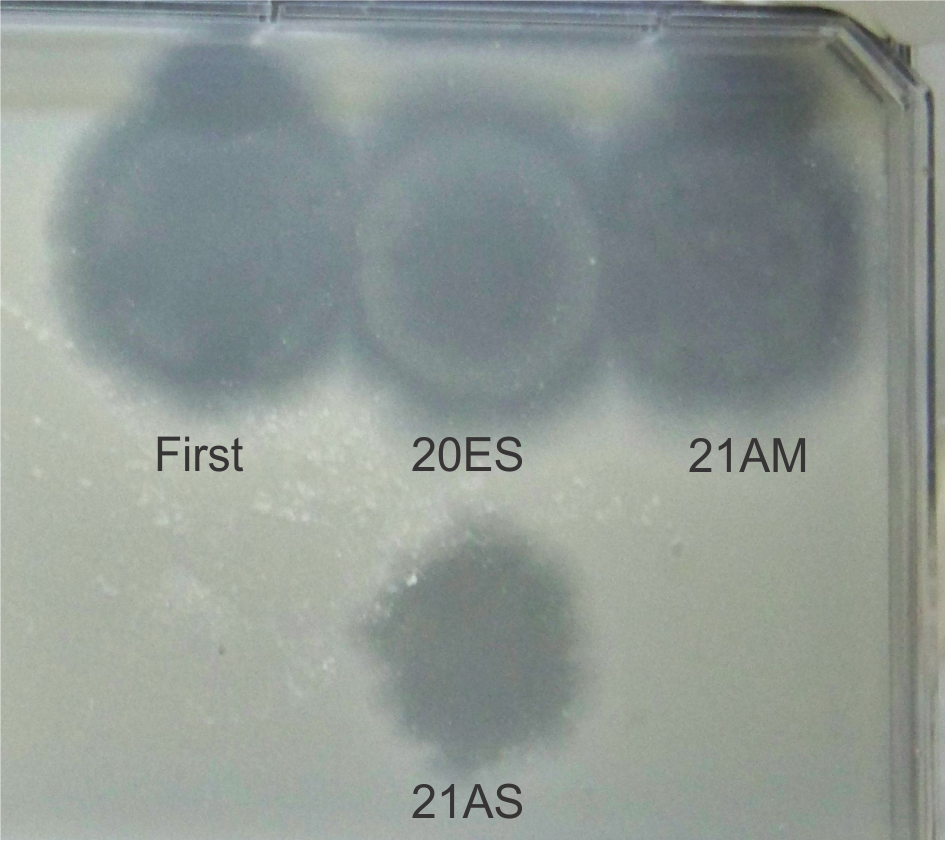

Supplement: Figure S3 — Four mycobacteriophages infect M. tuberculosis H37Rv. Aliquots containing 103 PFU of mycobacteriophages First, 20ES, 21AM and 21AS were spotted onto a lawn of M. tuberculosis H37Rv grown on 7H9ADS-Gly plates and incubated at 37°C for 10 days before visual inspection. (TIF) [file pone.0056384.s003.tif]
