# Supplementary material for: Analysis of Novel Mycobacteriophages Indicates the Existence of Different Strategies for Phage Inheritance in Mycobacteria
Source: PLoS One. 2013 Feb 28;8(2):e56384. doi: 10.1371/journal.pone.0056384 (PMC3585329; doi:10.1371/journal.pone.0056384)
Supplement: Table S1 — Oligonucleotides used to amplify the mycobacteriophage genomic ends. (DOCX) [file pone.0056384.s004.docx]

| **Oligonucleotide** | **Sequence (5´-3´)** |
| --- | --- |
| 32HC-Fw | GCTCTGACCTGCAATGATG |
| 32HC-Rv | ATCAGACGCCGGATTCTCG |
| First-Fw | TAAGCTGCTCACTATCGCATCG |
| First-Rv | GTGTGGTAGCTCACGGATGAC |
| 41HC-Fw | AAGCGTCACCGGAATCGGTC |
| 41HC-Rv | TCCTCGAATGGGAGTTCATC |
| 40AC-Fw | CGGCCTCGAGCATCCTTG |
| 40AC-Rv | TCCGGTGCCTTGACATCTCC |
| CRB1-Fw | CATTGTGGCCGGCTAATGCG |
| CRB1-Rv | CCGACTCTCCGATCGACTTG |
| 20ES-Fw | TCGAGCATGCGTGCTGTTAG |
| 20ES-Rv | GACGACGACCACAGCATGAG |
| Jolie2-Fw | TCTTGGCCGGCTTGGGAG |
| Jolie2-Rv | GCGAACACGGCAACTGGATG |

**Table S1. Oligonucleotides used to amplify the mycobacteriophage genomic ends.**
