# Supplementary material for: Analysis of Novel Mycobacteriophages Indicates the Existence of Different Strategies for Phage Inheritance in Mycobacteria
Source: PLoS One. 2013 Feb 28;8(2):e56384. doi: 10.1371/journal.pone.0056384 (PMC3585329; doi:10.1371/journal.pone.0056384)
Supplement: Table S2 — Cation dependence of mycobacteriophage infection of M. smegmatis mc2 155. Phage titre assays were performed using Middlebrook 7H9-Gly agar. An aliquot of a culture of M.smegmatis, used as indicator strain, was included on a top agar made with the same medium. In all cases both medium were either devoid of calcium and magnesium salts or supplemented with 10 mM of each cation of their combination. The same conditions were used during the adsorption step. Aliquots containing 1/10 dilutions of each phage were spotted on the top agar. The minimum PFU required to produce detectable lysis was visually determined. (DOCX) [file pone.0056384.s005.docx]

| **Phage** | **Minimum PFU required for visible lysis** | | | |
| --- | --- | --- | --- | --- |
|  | **No cation** | **Mg ^2+ (a)^** | **Ca^2+ (a)^** | **Ca^2+-^Mg ^2+ (a)^** |
| 19ES | 10 | 10 | 10 | 10 |
| Jolie1 | 10^3^ | 10 | 10 | 10 |
| Hosp/Mine | 10^5^ | 10 | 10 | 10 |
| Bahia1/21AS/CRB2/39HC | 10^7^ | 10 | 10 | 10 |
| Bahia2/Jolie2/40BC/First/32HC | 10^5^ | 10 | 10 | 10 |
| 21AM | 10^7^ | 10^5^ | 10 | 10 |
| 20ES/40AC/41HC | 10^5^ | 10^3^ | 10 | 10 |
| CRB1 | 10^5^ | 10^5^ | 10 | 10 |

**Table S2.** **Cation dependence of mycobacteriophage infection of *M. smegmatis* mc^2^ 155.** Phage titre assays were performed using Middlebrook 7H9-Gly agar. An aliquot of a culture of *M.smegmatis*, used as indicator strain, was included on a top agar made with the same medium. In all cases both medium were either devoid of calcium and magnesium salts or supplemented with 10 mM of each cation of their combination. The same conditions were used during the adsorption step. Aliquots containing 1/10 dilutions of each phage were spotted on the top agar. The minimum PFU required to produce detectable lysis was visually determined.
