# Supplementary material for: Analysis of Novel Mycobacteriophages Indicates the Existence of Different Strategies for Phage Inheritance in Mycobacteria
Source: PLoS One. 2013 Feb 28;8(2):e56384. doi: 10.1371/journal.pone.0056384 (PMC3585329; doi:10.1371/journal.pone.0056384)
Supplement: Table S3 — Characteristics of novel mycobacteriophages. (/) in the “cation requirement” column means no preferences for Ca2+ or Mg2+; Mtb, M. tuberculosis H37Rv; BCG, M. bovis var BCG strain Pasteur; Msmeg, M. smegmatis mc2155; D, sequence deposited in GenBank; AS, assembled sequence; PAS, partially assembled sequence. (DOC) [file pone.0056384.s006.doc]

| **Phage** | **Life cycle** | **Isolation**  **temperature** | **Host range** | **Cation**  **requirement** | **Status of bioinformaticanalysis** |
| --- | --- | --- | --- | --- | --- |
| First | temperate | 37ºC | Mtb/BCG/Msmeg | Ca^2+^/Mg^2+^ | D |
| 41HC | temperate | 37 ºC | Msmeg | Ca^2+^ | AS |
| 40AC | lytic | 37 ºC | Msmeg | Ca^2+^ | AS |
| 20ES | temperate | 37 ºC | Mtb/BCG/Msmeg | Ca^2+^ | AS |
| CRB1 | temperate | 37 ºC | Msmeg | Ca^2+^ | AS |
| Bahia1 | lytic | 37 ºC | Msmeg | Ca^2+^/Mg^2+^ |  |
| 32HC | temperate | 37 ºC | Msmeg | Ca^2+^/Mg^2+^ | AS |
| 21AM | temperate | 37 ºC | Mtb/BCG/Msmeg | Ca^2+^ | PAS |
| 21AS | temperate | 37 ºC | Mtb/BCG/Msmeg | Ca^2+^/Mg^2+^ | PAS |
| 19ES | temperate | 37 ºC | Msmeg | none |  |
| 40BC | lytic | 30 ºC | Msmeg | Ca^2+^/Mg^2+^ | AS |
| Jolie2 | lytic | 30 ºC | Msmeg | Ca^2+^/Mg^2+^ | AS |
| Hosp | lytic | 30 ºC | Msmeg | Ca^2+^/Mg^2+^ | AS |
| Jolie1 | lytic | 30 ºC | Msmeg | Ca^2+^/Mg^2+^ | AS |
| 39HC | lytic | 30 ºC | Msmeg | Ca^2+^/Mg^2+^ | AS |
| Bahia2 | lytic | 30 ºC | Msmeg | Ca^2+^/Mg^2+^ |  |
| Mine | lytic | 30 ºC | Msmeg | Ca^2+^/Mg^2+^ | AS |
| CRB2 | lytic | 30 ºC | Msmeg | Ca^2+^/Mg^2+^ |  |

**Table S3. Characteristics of novel mycobacteriophages.**

(/) in the “cation requirement” column means no preferences for Ca^2+^ or Mg^2+^; Mtb, *M. tuberculosis* H37Rv; BCG, *M. bovis* var BCG strain Pasteur; Msmeg, *M. smegmatis* mc^2^155; D, sequence deposited in GenBank; AS, assembled sequence; PAS, partially assembled sequence.
