# Supplementary material for: Analysis of Novel Mycobacteriophages Indicates the Existence of Different Strategies for Phage Inheritance in Mycobacteria
Source: PLoS One. 2013 Feb 28;8(2):e56384. doi: 10.1371/journal.pone.0056384 (PMC3585329; doi:10.1371/journal.pone.0056384)
Supplement: Table S4 — Annotation of ORFs in the genome of mycobacteriophage First. Coordinates and putative functions of First are indicated. A tRNA:tRNA-Gln(ctg) is encoded at coordinates 4425–4503 bp but not listed.Direction of transcription, F, forward (leftwards); R, reverse (rightwards). (DOCX) [file pone.0056384.s007.docx]

| **ORF** | **F/R** | **Position** | **Size(aa)** | **Nearest homologue (% identity)** | **Putative function** |
| --- | --- | --- | --- | --- | --- |
| 1 | F | 569-1336 | 255 | Redrock gp1 (88%) | unknown |
| 2 | F | 1591-2184 | 197 | Turbido gp3 (81%) | unknown |
| 3 | F | 2181-2480 | 100 | Turbido gp 4(85%) | unknown |
| 4 | F | 2483-2779 | 98 | Redrock gp4(100%) | HNH endonuclease |
| 5 | F | 2818-3288 | 157 | Turbido gp6(97%) | unknown |
| 6 | F | 3375-4340 | 322 | Turbido gp7(87%) | Minor tail subunit |
| 8 | F | 4532-4690 | 52 | Trixie gp11(92%) | unknown |
| 9 | F | 4690-5988 | 432 | Trixie gp12(84%) | Lysin A |
| 10 | F | 5985-6446 | 153 | Turbido gp12 (86%) | Holin |
| 11 | F | 6443-7420 | 325 | Trixie gp14(89%) | Lysin B |
| 12 | F | 7443-9233 | 596 | Turbido gp14(97%) | Terminase |
| 13 | F | 9230-10690 | 486 | Turbido gp15(89%) | Portal |
| 14 | F | 10720-11583 | 287 | Turbido gp16(85%) | Capsid maturation protease |
| 15 | F | 11599-12150 | 183 | Pukovnik gp17(83%) | Scaffold protein |
| 16 | F | 12180-13136 | 318 | Trixie gp19 (98%) | Major capsid |
| 17 | F | 13206-13382 | 58 | Turbido gp19 (72%) | unknown |
| 18 | F | 13386-13763 | 125 | Turbido gp20(94%) | unknown |
| 19 | F | 13760-13954 | 64 | Pukovnik gp21(87%) | unknown |
| 20 | F | 13951-14319 | 122 | Turbido gp22 (98%) | unknown |
| 21 | F | 14319-14654 | 111 | Trixie gp24 (95%) | unknown |
| 22 | F | 14664-15095 | 143 | Redrock gp25(88%) | unknown |
| 23 | F | 15114-15710 | 198 | Turbido gp25(93%) | Major tail subunit |
| 24 | F | 15760-16230 | 156 | Turbido gp26(93%) | Tail assembly chaperone |
| 25 | F | 15760-16652 | 297 | Turbido gp27(86%) | Tail assembly chaperone |
| 26 | F | 16642-19185 | 847 | Turbido gp28(88%) | Tapemeasure protein |
| 27 | F | 19219-20229 | 336 | D29 gp27(91%) | Minor tail subunit |
| 28 | F | 20226-21998 | 590 | Turbido gp30(94%) | Minor tail subunit |
| 29 | F | 22076-22519 | 147 | Turbido gp31 (94%) | unknown |
| 30 | F | 22516-22857 | 113 | Bxz2 gp31(55%) | unknown |
| 31 | F | 22858-25263 | 801 | Turbido gp33(61%) | Minor tail subunit |
| 32 | F | 25337-25531 | 64 | Packman gp34 (60%) | unknown |
| 33 | F | 26162-26722 | 186 | Redrock gp37 (86%) | ParA |
| 34 | F | 26715-26993 | 92 | Redrock gp38(78%) | ParB |
| 35 | R | 27171-27341 | 56 | No database match |  |
| 36 | R | 27338-27556 | 72 | Trixie gp38(65%) | unknown |
| 37 | R | 27566-27751 | 61 | Turbido gp39(54%) | unknown |
| 38 | R | 27748-28125 | 125 | Turbido gp40(88%) | unknown |
| 39 | R | 28103-28390 | 95 | Turbido gp41 (85%) | unknown |
| 40 | R | 28387-28587 | 66 | Turbido gp42(55%) | unknown |
| 41 | R | 28571-28870 | 99 | Redrock gp44(54%) | unknown |
| 42 | R | 28870-29103 | 77 | Redrock gp43(84%) | unknown |
| 43 | R | 29100-29321 | 73 | Trixie gp43(92%) | unknown |
| 44 | R | 29355-31142 | 535 | Turbido gp45(92%) | DNA polymerase I |
| 45 | R | 31151-31504 | 117 | Jeffabunny gp47(57%) | unknown |
| 46 | R | 31501-31689 | 62 | Turbido gp47 (92%) | unknown |
| 47 | R | 31689-31889 | 66 | No database match |  |
| 48 | R | 31889-32311 | 140 | Redrock gp49(79%) | HTH binding protein |
| 49 | R | 32308-32517 | 69 | Turbido gp49(91%) | unknown |
| 50 | R | 32510-32758 | 82 | *Mycobacterium rhodesiae* hypothetical protein(64%) | unknown |
| 51 | R | 32760-33488 | 242 | Turbido gp50(92%) | ThyX-like (Thymidylate synthase) |
| 52 | R | 33565-34110 | 181 | Trixie gp48(73%) | unknown |
| 53 | R | 34107-36164 | 685 | Trixie gp49(89%) | Ribonucleotide reductase |
| 54 | R | 36164-36367 | 67 | TA17A hypotethical protein(91%) | unknown |
| 55 | R | 36364-36546 | 60 | Turbido gp54 (88%) | unknown |
| 56 | R | 36543-37304 | 253 | *Mycobacterium rhodesiae* hypothetical protein (52%) | unknown |
| 57 | R | 37301-37453 | 50 | Trixie gp53(84%) | unknown |
| 58 | R | 37457-38227 | 257 | Turbido gp57(95%) | Metallophosphoesterase |
| 59 | R | 38224-38547 | 107 | Redrock gp59 (89%) | unknown |
| 60 | R | 38544-34621 | 25 | Redrock gp60 (92%) | unknown |
| 61 | R | 38621-39106 | 161 | Turbido gp60(98%) | DNA primase |
| 62 | R | 39081-39473 | 130 | Trixie gp58(82%) | DNA primase |
| 63 | R | 39542-39676 | 44 | Trixie gp59(98%) | unknown |
| 64 | R | 39702-40175 | 157 | Turbido gp63(97%) | Endonuclease VII |
| 65 | R | 40141-40254 | 37 | Gladiator gp62(55%) | unknown |
| 66 | R | 40251-41090 | 279 | Turbido gp65(91%) | Esterase/lipase |
| 67 | R | 41087-41317 | 76 | Pukovnik gp63(88%) | unknown |
| 68 | R | 41465-41764 | 99 | *Pseudoxanthomonas suwonensis* hypothetical protein (34%) | HTH binding protein |
| 69 | R | 41792-42598 | 268 | Turbido gp69(95%) | DnaB like Helicase |
| 70 | R | 42609-42737 | 42 | No database match | unknown |
| 71 | R | 42734-42973 | 79 | Turbido gp71 (92%) | unknown |
| 72 | R | 43002-43196 | 64 | Peaches gp64 (94%) | unknown |
| 73 | R | 43193-43417 | 74 | Turbido gp73(84%) | unknown |
| 74 | R | 43466-44362 | 298 | Turbido gp74(95%) | RecB like nuclease |
| 75 | R | 44359-44772 | 137 | TA17A hypotethical protein(59%) | unknown |
| 76 | R | 44828-45400 | 190 | TA17A repressor protein(79%) | Repressor |
| 77 | R | 45638-45805 | 55 | Alma gp76(57%) | unknown |
| 78 | R | 45943-46101 | 52 | Trixie gp77 (78%) | unknown |
| 79 | R | 46103-46357 | 84 | Turbido gp79(76%) | unknown |
| 80 | R | 46357-46674 | 105 | Turbido gp80 (78%) | unknown |
| 81 | R | 46671-46943 | 90 | Turbido gp81(45%) | unknown |
| 82 | R | 46940-47167 | 75 | Redrock gp81 (99%) | unknown |
| 83 | R | 47176-47298 | 40 | Che12 gp85 (75%) | unknown |
| 84 | R | 47298-47621 | 107 | Turbido gp84(66%) | unknown |
| 85 | R | 47618-48376 | 252 | Redrock gp84 (87%) | unknown |
| 86 | R | 48376-48510 | 44 | Turbido gp86 (98%) | unknown |
| 87 | R | 48521-48829 | 102 | Turbido gp87(73%) | unknown |
| 88 | R | 48882-49307 | 141 | Redrock gp88 (87%) | unknown |
| 89 | R | 49328-49681 | 117 | Redrock gp89 (87%) | unknown |
| 90 | R | 49678-49845 | 55 | Redrock gp90(91%) | unknown |
| 91 | R | 49842-50042 | 66 | Turbido gp92(100%) | unknown |
| 92 | R | 50042-50335 | 97 | Redrock gp92(69%) | unknown |
| 93 | R | 50335-50622 | 95 | Trixie gp92(75%) | unknown |
| 94 | R | 50631-51341 | 236 | Trixie gp93 (93%) | unknown |
| 95 | R | 51372-51452 | 26 | EricB gp99(84%) | unknown |
| 96 | R | 51477-51767 | 96 | Turbido gp95(91%) | unknown |

**Table S4. Annotation of ORFs in the genome of mycobacteriophage First.** Coordinates and putative functions of First are indicated. A tRNA:tRNA-Gln(ctg) is encoded at coordinates 4425-4503bp but not listed..Direction of transcription, F, forward (leftwards); R, reverse (rightwards).
